# Exploiting Routine Clinical Measures to Inform Strategies for Better Hearing Performance in Cochlear Implant Users

**DOI:** 10.3389/fnins.2018.01048

**Published:** 2019-01-15

**Authors:** Alan P. Sanderson, Edward T. F. Rogers, Carl A. Verschuur, Tracey A. Newman

**Affiliations:** ^1^Institute of Sound and Vibration Research, Faculty of Engineering and the Environment, University of Southampton, Southampton, United Kingdom; ^2^Institute for Life Sciences and Optoelectronics Research Centre, University of Southampton, Southampton, United Kingdom; ^3^Auditory Implant Service, Faculty of Engineering and the Environment, University of Southampton, Southampton, United Kingdom; ^4^Clinical Neurosciences, Institute for Life Sciences, Faculty of Medicine, University of Southampton, Southampton, United Kingdom

**Keywords:** cochlear implant – neuroprosthesis, clinical monitoring and alerting, foreign body response, cochlear implant – impedance telemetry, hearing impairment

## Abstract

Neuroprostheses designed to interface with the nervous system to replace injured or missing senses can significantly improve a patient’s quality of life. The challenge remains to provide implants that operate optimally over several decades. Changes in the implant-tissue interface may precede performance problems. Tools to identify and characterize such changes using existing clinical measures would be highly valuable. Modern cochlear implant (CI) systems allow easy and regular measurements of electrode impedance (EI). This measure is routinely performed as a hardware integrity test, but it also allows a level of insight into the immune-mediated response to the implant, which is associated with performance outcomes. This study is a 5-year retrospective investigation of MED-EL CI users at the University of Southampton Auditory Implant Service including 176 adult ears (18–91) and 74 pediatric ears (1–17). The trend in EI in adults showed a decrease at apical electrodes. An increase was seen at the basal electrodes which are closest to the surgery site. The trend in the pediatric cohort was increasing EI over time for nearly all electrode positions, although this group showed greater variability and had a smaller sample size. We applied an outlier-labeling rule to statistically identify individuals that exhibit raised impedance. This highlighted 14 adult ears (8%) and 3 pediatric ears (5%) with impedance levels that deviated from the group distribution. The slow development of EI suggests intra-cochlear fibrosis and/or osteogenesis as the underlying mechanism. The usual clinical intervention for extreme impedance readings is to deactivate the relevant electrode. Our findings highlight some interesting clinical contradictions: some cases with raised (but not extreme) impedance had not prompted an electrode deactivation; and many cases of electrode deactivation had been informed by subjective patient reports. This emphasizes the need for improved objective evidence to inform electrode deactivations in borderline cases, for which our outlier-labeling approach is a promising candidate. A data extraction and analysis protocol that allows ongoing and automated statistical analysis of routinely collected data could benefit both the CI and wider neuroprosthetics communities. Our approach provides new tools to inform practice and to improve the function and longevity of neuroprosthetic devices.

## Introduction

Neuroprosthetics is a rapidly developing and profoundly important area of medical science and engineering. Substantial progress in this field, owing to improvements in biomaterials, electronics and computer science, presents opportunities to manage sensory and motor deficits that were previously untreatable. Neuroprosthetic interfaces of the central or peripheral nervous system share three common design objectives; selectivity of stimulation/recording to supplement function, bio-compatibility, and long-term reliability. Despite their differences in target tissue, size and function they all face the same challenges of longevity. Device wear and tear and the biological response to the device such as fibrosis are currently major limiting factors of efficacy in neuroprosthetics (Adewole et al., 2017). Although the micro-environments of the central and peripheral nervous system exhibit specific chemical and cellular profiles, the broad challenges are universal and are driving the need for improved understanding of the tissue-implant interactions.

Cochlear implants (CIs) are the most common and successful sensory neuroprosthetic device with almost 600,000 recipients worldwide (Ear Foundation, 2016). They enable people with severe and profound deafness to hear speech, music and environmental sound (Wilson and Dorman, 2008). They make ideal models for neuroprosthetic research because their performance can be measured both subjectively and objectively: CI users can describe their hearing experience to clinicians and researchers who can then remotely measure hardware performance *in-situ*. The most common cause of deafness is loss or damage to the hair cells in the cochlea, meaning that they cannot convert vibrations in the air into electrical signals for the brain to process. CIs collect sound through an external microphone, convert it to electrical signals, and directly stimulate the auditory nerve with these signals, bypassing the normal hearing mechanism within the outer, middle and inner ear. The device delivers a sequence of current pulses, similar to those generated by the biological hearing apparatus, through a platinum multi-electrode array positioned in the cochlea. The signals from the auditory nerve are then interpreted as for normal biological hearing, by processing in the central auditory pathways of the brain. In many cases this affords 100% speech recognition for the implant user when listening in favorable acoustic conditions (Gifford et al., 2008).

**FIGURE 1:**
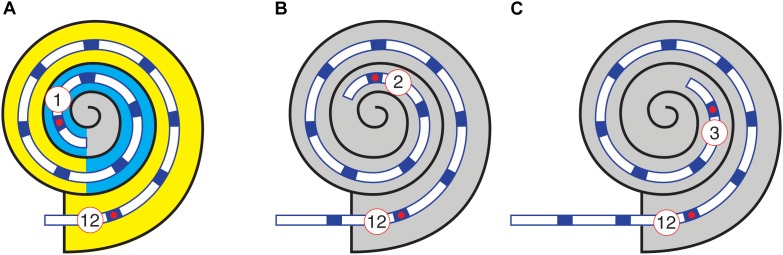
Example corrections of electrode number to account for extra-cochlear electrodes. Schematic represents the MED-EL Standard electrode array. **(A)** Full insertion (720°). **(B)** 1 extra-cochlear electrode. **(C)** 2 extra-cochlear electrodes. In **(A)**, the three turns of the cochlea are indicated by color: yellow, base; cyan, middle; gray, apex.

The cochlea consists of a bone encased membranous spiral containing the sensory apparatus of hearing and its supporting structures, which are essential for sensory transduction and homeostasis. The scalae of the cochlea are three tube-like chambers projecting through the spiral: the scala tympani, the scala media and scala vestibuli. The electrode is usually surgically inserted into the scala tympani, in close proximity to the spiral ganglion neurons (SGNs). The average total length of the cochlear spiral is 42 mm and the total length of the first complete turn is 22.6 mm (Rask-Andersen et al., 2011). The majority of human cochleae have between 2.5 and 2.75 turns (Biedron et al., 2009). For ease of reference, these turns are conventionally denoted base, middle and apex, from the largest to the smallest (Rask-Andersen et al., 2012) (Figure 1A).

Since the widespread introduction of CIs in the 1980s, there have been several refinements to the technology and related health policy. Improvements in hardware manufacture, signal processing strategies, surgical techniques and the relaxation of CI candidacy criteria have all contributed to better clinical outcomes, including preservation of residual hearing (Nguyen et al., 2016), improved speech recognition (Wilson and Dorman, 2008) and fewer device related adverse events (Causon et al., 2013). Despite these improvements, however, some users still experience poor or declining speech recognition, poor sound quality and stimulation of non-auditory sensations. In around 2% of cases, additional surgery is needed to explant and replace the CI. The explanted device is tested, and if hardware failure and surgical complications are excluded, a “soft failure” is diagnosed (Balkany et al., 2005). As hardware has improved, these soft failures, or idiopathic cases, have become relatively more common (Causon et al., 2013), and research is clearly needed to better understand how individual biology, and in particular the immune system, interacts with the neuroprosthesis to drive these adverse events. Conventional counts of soft failures only record those devices which perform badly enough to need surgical removal and not those that underperform, and so will necessarily under-estimate the influence of these biological factors.

Cochlear implants, like any bio-implant, stimulate an inflammatory response, which culminates in the encapsulation of the prostheses, in a sheath of fibrotic or scar tissue (Anderson et al., 2008). Currently, CIs are constructed from a silicone carrier and platinum electrodes. A common type of medical grade silicone, polydimethylsiloxane (PDMS) is quite well understood and is used in many bio-implants including breast implants (Hillard et al., 2017), cardiac pacemakers and spinal cord stimulators which help patients with chronic pain and to manage incontinence (Hassler et al., 2011). As well as the materials themselves, though, the tissue response is modulated by electrode microscopic surface topography and chemical composition (Christo et al., 2015). It seems that tissue growth in response to CI is inevitable (Li et al., 2007) although the nature and extent of the response is somewhat variable across individuals (Fayad et al., 2009).

Although fibrosis can foul the implant and impair its function, it is also beneficial in mechanically fixing the array within the cochlea. This helps create a seal to prevent both loss of perilymph and infiltration of bacteria from the middle ear (Stöver and Lenarz, 2009). A healthy inflammatory response to an injury comprises successive waves of pro- and anti-inflammatory chemokines and cytokines, controlled cellular migration to the wound site, with eventual resolution of inflammation and controlled apoptosis of recruited cells accompanied by wound repair and remodeling. In the case of implanted biodevices, the immune system reacts to the acute surgical trauma as well as the protracted exposure to the implanted biomaterials. Initially, the inflammatory response is characterized by exudation of fluid and plasma proteins from the circulation together with active infiltration of neutrophils to the surgical wound site. Proteins including fibrin are rapidly adsorbed onto the implanted biomaterial to form a provisional matrix that attracts macrophages, which can fuse to form multi-nucleated giant cells. Macrophages contribute to the fibrotic capsule by releasing cytokines that attract fibroblasts and stimulate them to secrete collagen.

The inflammatory process leads to the commonly described tissue reaction to a CI: a tightly packed layer of fibroblasts and collagen with occasional macrophages surrounding the electrode array (Grill and Thomas Mortimer, 1994). In the majority of cases, this tissue state remains stable over time. However, in some instances there is tissue hypertrophy, or extensive fibrosis and bone formation, which hinders the function of the electrode. Lim et al. (2011) found that pathological foreign body reactions (FBR) requiring revision surgery are rare. However, evidence from post-mortem temporal bones suggests that the characteristic indicators of FBR such as foreign body giant cells are more common than expected (Nadol et al., 2014; Seyyedi and Nadol, 2014). This highlights the potential for sub-clinical FBR, which does not reach soft-failure but is clearly detectable to post-mortem histological analysis. The complex reaction to CI often also includes new bone formation (osteogenesis) (Somdas et al., 2007). Osteogenesis appears more detrimental to implant performance than fibrosis and is associated with reduced speech discrimination scores, (Kamakura and Nadol, 2016) and an effective reduction in dynamic range of stimulus current (Kawano et al., 1998). It is therefore crucial to understand the transition from a healthy short-lived tissue response to a chronic or spontaneous over-exuberant response.

Studies of donated temporal bones from CI users have shown that intra-cochlear location can significantly affect tissue development after CI implantation. The basal, high-frequency region of the cochlea exhibits significantly greater fibrosis and osteogenesis, and poorer survival of both hair cells and peripheral projections of SGNs (Fayad et al., 2009). Histological analysis identifies greater numbers of giant cells and lymphocytes at the cochleostomy site than at the mid and apical regions of the cochlea (Seyyedi and Nadol, 2014). In addition to the consistent pattern of basal tissue hypertrophy, some individuals also exhibit fibrosis that extends along the full length of the electrode array and beyond (Somdas et al., 2007). There is evidence that the volume of new tissue correlates with the level of damage to the lateral wall (Li et al., 2007) and other structures including the basilar membrane (Kamakura and Nadol, 2016). While this data is intriguing, and clearly points to the importance of the biological response to the implant, it is limited to post-mortem studies, meaning that the majority of the data is collected after long-term implantation. This means it cannot be used to interpret performance fluctuations, and does not give us the early warning of soft failure that would be so useful in the clinic.

A readily available, non-invasive, clinical measure from a CI is electrode impedance (EI) telemetry (Hughes et al., 2001). EI describes the ease with which electrical current flows through and between implanted electrodes. The CI stimulator delivers a current pulse that flows through the platinum electrodes of the CI and into the ionic environment of the cochlear tissue. This pulse must be calibrated so that it delivers sufficient of charge to stimulate the SGN, without damaging the tissue. High EI means the implant must deliver a higher voltage to maintain the delivered charge. This has two undesirable effects: it drains the battery of the device faster and, more importantly, it spreads the excitation across more SGN reducing frequency resolution, and in turn the quality of the perceived sound. In general, therefore, low EI makes it more likely that an implant performs well.

The EI is determined by delivering a low-level current pulse through the relevant electrode inputs on the CI and measuring the resulting voltage across the associated electrodes. It can be performed quickly in the clinic using a hardware interface that connects the implant to a computer via a transcutaneous link. In the clinic, EI telemetry is primarily used as an electrode integrity test. Open or short circuit faults (very high or very low impedances, respectively) can easily be diagnosed, which is useful to clinicians in deciding whether a given electrode should be activated. These faults are relatively common: Carlson et al. (2010) showed a 9% chance of either at least one open- or short-circuit fault in an implanted device.

Despite its primary role as an integrity check, EI is a continuous measure, which can provide much more information on the biology around the implant. A major factor in determining EI is the volume and composition of bulk tissue surrounding the implanted electrode array (Tykocinski et al., 2001). Clark (2003) recommends that EI levels should be monitored routinely as an indicator of cochlear tissue changes such as fibrosis and electrode surface roughening. In a study of chronic high-rate stimulation using cats, Xu et al. (1997) demonstrated that levels of fibrosis and presence of inflammatory cells were greatest in the cochleae that exhibited the greatest EI levels. Clark et al. (1995) found that EI was significantly correlated with the amount of tissue around the electrode contacts and cases where inflammatory cells were found in the tissue showed particularly high levels of EI.

The studies above show the value of EI as an indicator of tissue status, but initial studies also show that it may be useful for predicting patient outcomes. Electrodes that exhibit high impedance levels are associated with raised thresholds of auditory sensation and reduced dynamic range (Busby et al., 2002) which can be associated with poorer performance outcomes (Wolfe et al., 2013). EI increase and/or fluctuation are recognized as clinical indicators of soft-failure (Balkany et al., 2005). The onset of sudden changes in EI over time are correlated with marked loss of residual hearing in CI users (Choi et al., 2017).

Considering the potential value of monitoring and interpreting EI fluctuations, there is a surprising lack of consensus guidance on clinical utility of impedance telemetry, especially in light of its proven association with the immune-mediated tissue response. A number of authors have shown greater EI levels in the basal region of the cochlea compared to more apical locations. Jia et al. (2011) analyzed EI from 20 adult CI users and found higher levels at the basal position after 3 months that were maintained for the 36-month study duration. The pattern of raised EI at basal electrodes has been observed in other clinical CI studies (Hughes et al., 2001; Busby et al., 2002; Leone et al., 2017) and supports the temporal bone histology studies showing greater tissue growth in this region. These studies, which draw from cohort sizes ranging from 19 to 35 individuals, have generated useful preliminary evidence. However, a lack of larger study groups—ideally complete clinical caseloads—combined with the known inter-patient variability, is a major factor in the lack of clinical consensus. To date there is no published evidence of a clinical platform for systematic analysis of EI to produce normative models, against which individuals can be compared.

There is evidence that change in EI over time can serve as an indicator of the immune-mediated tissue response. Following surgical implantation of the CI electrode array, the tissue undergoes rapid changes attributable to the acute inflammatory response (Shepherd et al., 1994). This change manifests in a measurable increase in EI between implantation and the date of activation (Busby et al., 2002; Saunders et al., 2002). Several studies report a significant reduction in EI following commencement of electrical stimulation, which often plateaus over 1–3 months (Hughes et al., 2001; Henkin et al., 2006; Jia et al., 2011). After the initial stimulation-induced reduction, EI usually remains at a stable level in actively stimulated electrodes for several months (Henkin et al., 2003, 2006), while inactive electrodes show a steady increase over time (Dorman et al., 1992; Hughes et al., 2001).

The present study is a retrospective investigation of clinical data from an auditory implant service and demonstrates the untapped value in clinical recordings taken from neuroprostheses—in our case, CIs. As shown above, there is a pressing need to reduce the wide variance of outcomes and improve implant longevity, which will be substantially helped by improving observations of the CI-tissue interface. We describe sample-wide variability over 5 years. This view is not available through the clinical software, which prevents clinicians from easily identifying deviations from normal. We asked the question: what is the general trend of impedance change over time for different electrode positions? Based on previous evidence of tissue proliferation around the round window and hook region we predicted that the electrodes furthest from the base would show lower impedance with a downward trend over time. Next, we applied an upper threshold to identify individuals with raised impedance, statistically outside the main distribution but below the manufacturer’s “high impedance” warning level. We asked the questions: how many individuals exhibit significantly raised impedance levels? Of these, how many were identified with raised impedance at electrodes away from the base? These are particularly interesting cases to consider because no mechanism has been proposed for localized tissue proliferation away from the site of array insertion, i.e., cochleostomy or round window. This information could be used as early detection of unwanted inflammatory responses caused by the implant and its function rather than the surgery, which may go on to affect the CI interface and therefore longer-term performance. Clinical data review, like that proposed here, incurs a negligible burden on the CI user and minimal cost in both money and time.

## Materials and Methods

### Ethics Statement

This study was carried out in accordance with the recommendations of the University of Southampton Ethics Committee (UEC) and Faculty of Engineering and the Environment Ethics Committee (FEC). The protocol was approved by the FEC. All subjects gave written informed consent in accordance with the Declaration of Helsinki. [UEC Ethics ID: 17430].

### Participants

The study included 172 adult (176 adult ears) and 47 children (74 ears). Mean adult age was 58 years (18–91) and mean child age was 4.5 years (1–17). The patients included were implanted using either cochleostomy (approximately one third) or round window insertion (approximately two thirds). Data were collected from two sources within the University of Southampton Auditory Implant Service (USAIS); the clinical software database MED-EL Maestro and the local patient database.

### Electrode Characteristics

Study participants had previously received MED-EL Standard (*n* = 131), Flex-28 (96), Flex-24 (7), Flex-Soft (2), or Form24 (1) CI arrays. These are relatively long arrays enabling EI measures to be taken at a wide range of physical positions in the cochlea. For example, the Standard array has an active stimulation range of 26.4 mm, which is equivalent to two turns of the cochlea or an insertion angle of 720°. Each array carries 12 electrodes, each of which has either one or two exposed electrical contacts, depending on the array model. The effective electrode surface area for these MED-EL electrodes is 0.13–0.14 mm^2^.

### EI Data Acquisition

The main study aims were to describe the trends of EI in a large sample and highlight individuals who deviate from this. A single manufacturer and limited number of arrays were chosen to minimize the hardware variability with a view to focusing primarily on the soft or biological mechanisms for impedance evolution. Importantly, the method of voltage acquisition and impedance calculation varies significantly between manufacturers. The method used by the MED-EL telemetry system is shown in Supplementary Figure S1. The change in EI can be separated into two components; access resistance and polarization impedance. The latter reflects the physical properties of the electrode surface and is therefore affected by protein adsorption, surface area increase and localized ionic changes (Tykocinski et al., 2005; Newbold et al., 2010). The stimulation-induced EI reduction, which occurs rapidly following device activation, is dominated by this component (Newbold et al., 2014). Access resistance is known to reflect the bulk material around the electrode such as fluid, cells and tissue and is likely to change over longer time scales. Clinically available impedance telemetry does not allow the two components to be measured separately; however, using the MED-EL system allows both impedance components to be captured. Therefore, changes occurring over different time scales give some indication of the relative contribution of the two components. The impedance measurement is performed using monopolar, low-amplitude bi-phasic current pulses, similar to those used for stimulation via the device. Total impedance (*Z*_t_) can calculated using total voltage which is measured at the end of the current pulse (See Supplementary Figure S1). Total impedance comprises the developing polarization component (*Z*_p_) and the access resistance component (*R*_a_). EI is calculated as: *Z*_t_ = *V_t_*/*I*.

### EI Data Management

**FIGURE 2:**
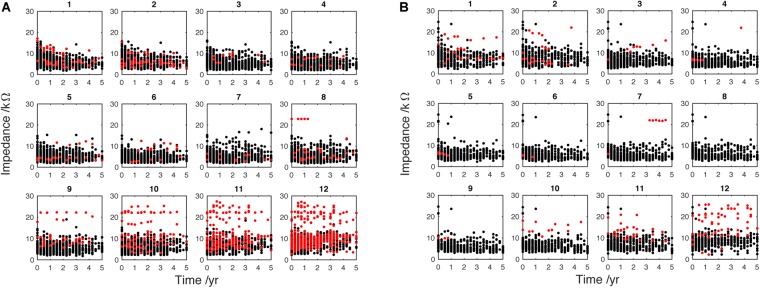
Electrode impedance (kΩ) measured over 5 years from implantation. **(A)** (adult, *n* = 176) and **(B)** (pediatric, *n* = 66) data are split into separate electrodes, from apical (1) to basal (12). Each dot represents the 3-month-average EI for one individual patient. The timeline for each patient begins with their respective device activation (time 0). Black dots, active electrodes; Red dots, deactivated electrodes. These data have been adjusted to correct for extra-cochlea position (see Figure 1).

**FIGURE 3:**
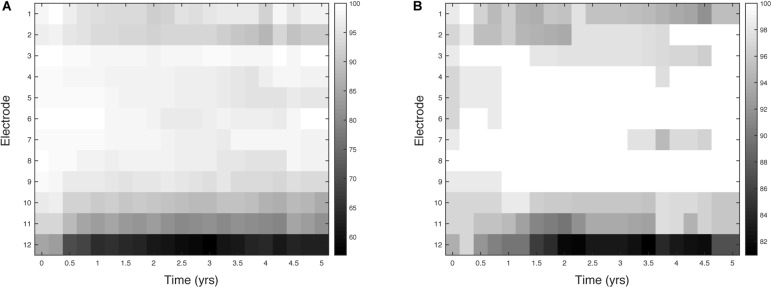
Percentage activation for 12 electrodes over 5 years. Each square represents a 3-month epoch for a given electrode. **(A)** (adult, *n* = 176) and **(B)** (pediatric, *n* = 66).

**FIGURE 4:**
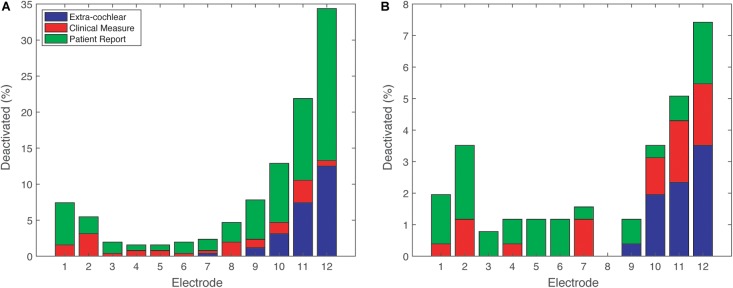
Reasons for deactivation across 12 electrodes. Patient Report (e.g., poor sound quality), Clinical Measure (e.g., impedance telemetry). **(A)** (adult, *n* = 176) and **(B)** (pediatric, *n* = 66).

**FIGURE 5:**
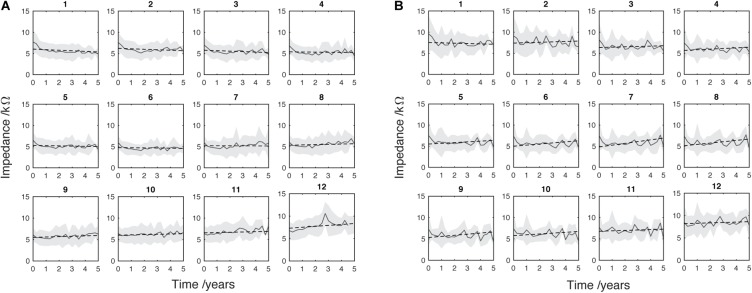
Mean EI (solid gray line) with SD (light gray shading). Regression line of least-squares (black dotted line) was fitted. **(A)** (adult, *n* = 176) and **(B)** (pediatric, *n* = 66) data are split into separate electrodes from apical (1) to basal (12). At this stage of analysis data from deactivated electrodes were removed and electrode number was corrected to account for basal extra-cochlear electrodes. The timeline for each patient begins with their respective device activation (time 0) and subsequent points represent 3-month intervals.

**FIGURE 6:**
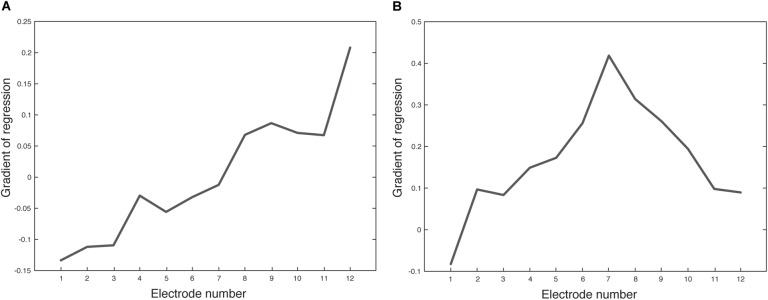
Gradient of regression lines (Figure 5) for each electrode. **(A)** (adult, *n* = 176) and **(B)** (pediatric, *n* = 66). Positive gradient values represent a trend of EI increase over time and negative gradient values represent a trend of EI decrease over time.

**FIGURE 7:**
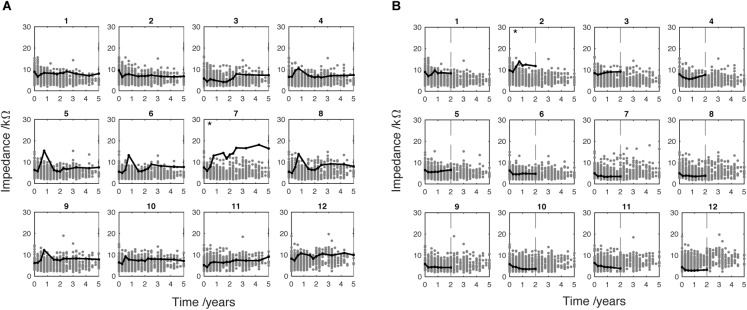
Two individual adult cases showing EI at active electrodes only. Case **(A)** shows 5 years CI use. Case **(B)** shows 2 years CI use indicated by vertical dotted line. Electrode marked by ^∗^ met the SEI criteria which indicates high EI compared to the sample distribution. These data have been adjusted to correct for extra-cochlea position (see Figure 1).

**FIGURE 8:**
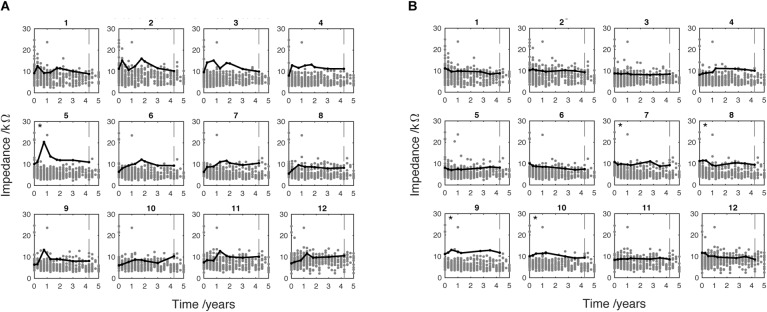
Two individual pediatric cases showing EI at active electrodes only. Case **(A)** shows 4.25 years CI use (indicated by vertical dotted line). Case **(B)** shows 4.25 years CI use (indicated by vertical dotted line). Electrode marked by ^∗^ met the SEI criteria which indicates high EI compared to the sample distribution. These data have been adjusted to correct for extra-cochlea position (see Figure 1).

Data were exported from MED-EL Maestro in Microsoft Access format. A custom database query was then used to return anonymized individual patients with their age at implant, implanted ear, date of birth, electrode activation status, electrode specific EI and corresponding date stamp. The difference between the date of implant and date stamp for each EI measurement was used to normalize data to a 0 date (day 0 is date of implantation) for each patient. Subsequent EI measurements were split according to the 12 individual electrodes and then averaged into 3-month time bins. All query results were exported in Microsoft spreadsheet format. MathWorks MATLAB (R2018a) was used to read data from excel spreadsheets and plot Figures 2–8.

### Deactivated Electrode Data Filtering

It is very common for CI users to have electrodes deactivated by clinicians. As discussed, several studies show an increasing EI in the absence of electrical stimulation. Therefore, to minimize the effect of this upward bias on the analysis, only data from actively stimulating electrodes (black dots in Figure 2) were included in analyses from Figure 5 onward; deactivated electrodes (red dots in Figure 2) were automatically removed from the analysis using a custom MATLAB script.

### Electrode Numbers Were Corrected for Extra-Cochlea Position

During surgery, it is common for the electrode array not to be fully inserted in the cochlea, meaning that electrodes (referred to by position along the array) may be shifted relative to the cochlear anatomy. We corrected for this effect to allow meaningful comparison of electrode positions between patients. Surgical records were interrogated to determine presence/number of extra-cochlear electrodes. The following correction was applied: correct electrode number = [original electrode number + number of extra cochlear electrodes; maximum of 12]. Figure 1 shows how this results in new electrode numbers being assigned to intra-cochlear electrodes. This does not allow for an estimation of insertion depth, but it does enable analysis of electrodes from “most basal” onward. This correction is applied to all data in Figures 2, 3, 5–8. The correction is not applied to Figure 4 (analysis of reasons for deactivation) as it would mask extra-cochlear deactivations.

### Statistical Analysis

The software program MathWorks MATLAB was used for data analysis. The adult and pediatric groups were analyzed separately. Least-squares linear regression lines were fitted to the average impedance data (Matlab polyfit) (Figure 5).

Using MATLAB, an outlier-labeling rule was applied to identify instances of raised EI (Figures 7, 8 and Supplementary Material)

$$T=Q_{u}+k\left(Q_{u}-Q_{l}\right)$$

(Hoaglin et al., 1986):

where *Q*_u_ and *Q*_l_ are the upper and lower quartiles, respectively, and *T* is the threshold for an outlier. The constant *k* was fixed at 2.2, equivalent to a 5% probability of any given measurement being an outlier, for the adult and pediatric sample sizes tested (Hoaglin and Iglewicz, 1987). Cases were highlighted as statistically raised EI (SEI) when the EI was greater than *T* in ≥2 time bins within the first 2 years of CI use. Current methods of “high impedance” detection are based on the upper limits of the stimulus delivery hardware for individual cases. Our new approach allows investigation of raised, but not extreme, levels of EI that would otherwise be considered sub-clinical.

Highlighted cases of SEI are split into “basal” (9–12) and “non-basal” (1–8) depending on the position of the electrode showing raised EI. Basal electrodes, which are nearest to the insertion site, are expected to show significantly stronger immune-mediated tissue development: previous studies show significantly greater EI corresponding to this region. A judgment was made to categorize electrodes that are likely to be in the hook region as “basal.” This is the straight region of the first cochlear turn, which extends 9 mm from the round window before it curves (Clark et al., 1990). The MED-EL Standard and Flex28 electrode arrays have contacts spaced at 2.2 and 1.9 mm, respectively (Med-El, 2013). This means that the basal portion of the array (electrodes 9–12) spans 8.8 and 7.6 mm for Standard and Flex-28 electrodes, respectively.

## Results

Data from 242 ears (176 adult and 66 pediatric) were included in the main analysis of EI changes over time. Figure 2 shows subplots representing 12 separate electrodes. The magnitude of EI is plotted against time from initial CI activation to 5 years later. Each single dot represents the average EI level for a single patient over 3 months. Impedance data measured from actively stimulating electrodes are indicated by black dots whereas data measured at deactivated electrodes are indicated by red dots. The subplots both show a large number of deactivated electrodes, particularly at the most apical and basal electrodes (1 and 12, respectively), the reasons for which are analyzed below. Figure 2A identifies a high number of deactivated basal electrodes for the adult population. Note that there are fewer dots at later time points, as not all patients had been using the device for the whole 5-year study period. The EI data were corrected to account for electrodes that were positioned outside the cochlea (see Section “Materials and Methods” and Figure 1). This was done to allow alignment of impedance data around an approximate physical position in the cochlea.

The proportion of deactivated electrodes in the population is shown in Figure 3. Deactivation is clearly most common in the most basal electrodes for both adults and children. The figure also shows an increasing number of deactivations over the first 1–2 years of CI use. The peak number of deactivations was higher in the adult group (Figure 3A) than the pediatric group (Figure 3B). Both groups had most deactivations at electrode 12, which can be seen as black at 2.25 years. At that epoch, only 60% of adult electrodes were active while 81% of pediatric electrodes were active. Electrode 11 showed the second highest number of deactivations for both groups. For example, 80% of adults had electrode 11 remaining active at 2.5, 3.25, 4 and 4.25 years. There was a slight increase in deactivations at the most apical electrodes compared to the mid-array for both adults and children. For example, adults had 88% of electrode 2 remaining active at 4 years. The children had 92% of electrode 1 remaining active at 4.5 years. A difference between the two groups was the mid-array electrodes were mostly active in the pediatric group, indicated by white area in Figure 3B. Although the adults were initially 100% active at electrodes 3 and 6, a few deactivations were made in the next 3-month epoch. In contrast, the children had 100% activation for the majority of the 5-year study period in electrodes 4, 5, 6 and 7.

The patterns of deactivation seen above are better understood in light of the clinical reasons for deactivation shown in Figure 4. Electrodes in the most basal portion of the array were deactivated because they were outside the cochlea (extra-cochlear). In the basal electrodes (9–12), extra-cochlear position accounted for about one third of the adult reasons (Figure 4A), and about half of the pediatric reasons (Figure 4B). The majority of deactivations, however, in the adult group were informed by the patient reports of their subjective experience, such as “poor sound quality” (See Supplementary Figure S2 for a complete list of deactivation reasons); there were relatively few deactivations owing to “Clinical Measures” which offer objective information. The percentage of subjective “Patient Report” reasons is highly likely to be biased by the age of the CI user: many of the children are very young and could not communicate their perception of sound. As shown in Figure 3, the children had significantly fewer deactivations overall.

Data points acquired at deactivated electrodes were removed at this stage of the analysis (red dots in Figure 2). Figure 5 shows the mean EI for the adults (Figure 5A) and the children (Figure 5B). Least-squares linear regression lines were fitted to the average impedance data (Matlab polyfit) for each electrode to show the trend of EI change over time. The adult group show a tendency for EI reduction at apical electrodes (negative slope), increase at basal electrodes (positive slope) and no change for mid electrodes. The pediatric group shows a different pattern of regression lines across the electrodes. All of the electrodes in this group, except electrode 1 show a positive slope. This suggests a difference in long-term EI evolution in children compared to adults, although the mean is more variable in this age group. This is probably caused by the lower overall sample size and fluctuation of sample size in each time window (i.e., by chance fewer individuals were seen in some 3-month epochs).

The data above indicates that EI changes over time in a way that varies with electrode position. We describe this EI change over time using a regression line for each electrode in Figure 5. The gradient of each line is plotted for each electrode in Figure 6. The adult group (Figure 6A) shows a positive relationship between gradient and electrode number. Each consecutive electrode shows a general increase in gradient with electrode number. The largely monotonic relationship between gradient and electrode fits the consensus in the literature and highlights the phenomena quite simply. Another observation is that the crossover point from EI reduction (negative gradient) to increase (positive gradient) is at electrode 7, which is roughly the middle of the electrode array. This shows that EI evolution varies from base to apex in a continuous fashion. The relationship between fit-line gradient and electrode number in the pediatric group (Figure 6B) shows that EI largely increases over the 5-year period for all electrodes except number 1. The increase is steepest at electrode 7. We note that the regression lines are an approximate linear fit and hence describe general trends. The pediatric sample shows a large degree of variability between timepoints because of the relatively low sample size and irregular frequency of clinical appointments. The peaks and troughs of mean EI cause some biasing of the fit lines so we have been conservative in our interpretation of differences between age groups.

In the adult group, 14 patients met the SEI criteria (8%): one in basal electrodes, three in both basal and non-basal and 10 in non-basal electrodes only. The case shown in Figure 7A was implanted with a standard electrode array and the clinical record did not include the hearing-loss etiology. The case shown in Figure 7B was implanted with a Flex28 electrode array and the clinical record showed head injury as the cause of hearing loss. Figure 7A shows an EI increase at electrode 7 over the 5 years of CI use. This electrode is highlighted by (^∗^) to indicate that EI level met the SEI criteria. A key observation is the difference in temporal development and absolute level of EI of this electrode compared to its immediate neighbors. This difference is unusual for non-basal electrodes where the EI is often mirrored in neighboring electrodes. The absolute EI level shown in Figure 7B is lower than Figure 7A although the SEI criteria have been met at electrode 2.

In the pediatric group, three cases met the SEI criteria (5%): two in non-basal and one in both basal and non-basal electrodes. Figure 8 shows the EI measurements taken from two pediatric cases. Each was found to meet the SEI criteria in one of two implanted ears. The black line shows that EI is greater in these cases than the other cases in the sample (gray dots) which are mostly clustered around or below 10 kΩ. The case shown in Figure 8A was implanted with a Flex-28 electrode. Clinical records show they were diagnosed with congenital hearing loss associated with Pendred syndrome. This case met the SEI criteria at electrode 5 (indicated by ^∗^). After the initial activation and tuning appointment, the EI increased relatively rapidly to peak around 1 year of CI use. A similarly sharp reduction is shown in the following 3-month period before EI plateau around 12 kΩ. This case shows a general tendency for raised EI over the duration of observed CI use. This is especially marked in electrodes 2, 3 and 4, although the level did not meet the criterion for SEI. The case shown in Figure 8B was fitted with a Standard electrode array. The clinical record showed a diagnosis of genetic mutation of the gene GJB2 (connexin26). The case shown in Figure 8B met the SEI criteria at electrode 7, 8, 9 and 10. Unlike the pattern shown in Figure 8A, the EI tracked a stable level across the period of use.

## Discussion

This retrospective study of clinical data from a large sample of MED-EL CI users showed population-level trends in EI across time and between cochlear regions, and also yielded a potential new approach to define EI outliers for whom further clinical action may need to be taken. The analysis showed that most adult electrode deactivations were made because of reported experiences rather than clinical measures such as neural-response telemetry or electrode-impedance telemetry. The population-based method of outlier detection used here offers an objective insight into intra-cochlear tissue status to inform decisions to deactivate electrodes. Ongoing challenges for neuroprostheses include biocompatibility and functional longevity (Adewole et al., 2017). Performance decrement, as contrasted with frank failure, is difficult to monitor and almost impossible to predict using current approaches. The consensus in the field of CI for clinical assessment of soft failure recommends a broad-spectrum approach. This includes patient interview, medical investigations such as X-ray imaging, audiological and hardware testing (Balkany et al., 2005). This relies on the CI user having well-established linguistic abilities. In children the consensus is that the clinician should record and interpret the user’s behaviors, although this has limited reliability (Moberly et al., 2013). The methods presented here allow deeper enquiry into the telemetry data that is already routinely gathered. Our results suggest that a minority of raised impedance cases can be detected in a population, which may aid triaging of patients, including those who can provide only limited verbal reports.

We describe the evolution of EI for adults and children at 12 electrodes along the MED-EL array. The measurement at the first (0 months) and second time points (3 months) identifies a drop in EI across all conditions. The drop is consistent with an increase in electrode surface area due to the electrolytic activity (Brummer and Turner, 1977), and/or clearance and reorganization of organic molecules, cells, tissues on and around the electrode (Marsella et al., 2014). The main observation in the adult group is EI growth at basal electrodes and EI reduction at apical electrodes. Growth in basal-electrode EI is likely to be caused by fibrosis and osteogenesis based on its slow evolution over time. Previous findings from a post-mortem study of cochleae from CI users have shown the levels of fibrotic and bone tissue to be greatest in the basal turn of the cochlea (Fayad et al., 2009). The magnitude of fibrosis is also correlated with the level of trauma caused by surgery (Richard et al., 2012). It is also possible that there are differences in capacity for inflammatory response in different regions of the cochlea, e.g., due to anatomical variations such as vasculature, nerve supply or cochlear-duct width.

We observed the trend that children show an increase in EI for all electrodes except electrode 1. This data shows more variability over time than the adults, possibly due to the lower number of cases analyzed. If a difference exists, the likely explanation is a difference in the chronic tissue response to surgery in children and adults (i.e., developmental stage) or differences in etiology among children vs. adults. Previous studies have shown increasing EI for basal, mid and apical electrodes in children compared to the adult group which only showed increase at the base (Hughes et al., 2001; Busby et al., 2002). Our data appear to support this although no formal age-group comparison was made. There is some published evidence of differences in hearing preservation between adults and children. One study showed a small trend toward better residual hearing in children (Zanetti et al., 2015), although another found no effect of age (Skarzynski et al., 2013). The findings of the present study suggest an increased growth of intra-cochlear tissue around the base that is particularly clear in the adult group.

The fact that gradual increases in basal impedance were observed is indicative of a slow proliferation of tissue indicative of immune-mediated fibrosis. Studies have shown that such reactions lead to structurally organized fibrotic tissue and bone (Li et al., 2007; Somdas et al., 2007), which would begin to emerge within the same timeframe as the impedance increase shown here, i.e., months to years. It should be noted that the exclusion of deactivated (mainly basal electrodes) would suggest that our findings under-estimate the extent of basal tissue growth. The data shows individual variability in EI, which may reflect surgical approach, age, etiology, noise exposure or other factors. The cases included were implanted using either cochleostomy (approximately one third) or round window insertion (approximately two thirds). No formal assessment of surgical approach and its impact on EI was carried out. Evidence shows that this variable has no significant effect on EI or listening performance for phonemes or sentences (Cheng et al., 2018).

We have limited understanding of the wide variability in performance and outcomes for CI users. A wealth of evidence suggests that the biological response to the implant is pivotal to its long-term functionality. It is possible to measure the response using impedance telemetry, although the currently available tools are limited to detection of extreme high or low EI levels. In order to address this, we applied a statistical method of outlier-labeling to detect cases of raised impedance (SEI). This is distinct from the absolute threshold used by the MED-EL and other manufacturers, which serves to highlight high and low impedances that are extreme enough to prevent normal current delivery. These cases mostly indicate hardware faults and extra-cochlear electrode position. The cost of using high threshold methods for detecting raised impedance is the relative insensitivity to biological perturbations associated with EI changes below 20 kΩ. Our technique could be validated by measuring CI performance following customization of processor maps where electrodes with SEI levels are deactivated. If validated, this would provide a quicker and more clinically useful method to guide electrode deactivation as compared with more challenging and time-consuming methods based on psychophysical measurements proposed in the literature: Mathew et al. (2017) and Zhou (2017). Further work to determine any correlation between the sorts of psychophysical methods proposed by these authors and the proposed outlier-EI values would help to further validate this approach.

The long-term pattern of change of EI in those individuals identified as outliers may inform the underlying mechanism. Results show EI increase at discrete electrodes, some developing slowly over the 5-year study period. In several cases (13 of 14) the SEI criteria was met at non-basal electrodes, which is counter to the model that the tissue development driven by inflammation is most prevalent at the base near the site of array implantation (Richard et al., 2012; Bas et al., 2015). In some cases, gradual EI differences are specific to particular non-basal electrodes. For example, electrode 7 in Figure 7A shows a pronounced example of EI increase that develops slowly over many months. In most cases, this was limited to one, or at most, very few electrodes, which suggests the change is driven by spatially localized factors. No hardware malfunctions were detected, and the electrode remained actively stimulated for the duration of the studied time period. One possible explanation is the presence of a spatially discrete trigger of inflammation such as mechanical trauma. This might have occurred during surgery as the electrode array tip passed through this region of the cochlear duct causing an abrasion, as lateral wall damage is known to elicit fibrotic changes (Li et al., 2007). To further understand the cause of raised but not “open-circuit” EI in particular electrode regions, the ability to cross-reference with newer and more sensitive imaging methods (Aschendorff, 2011) could also lead to a greater understanding of whether localized surgical trauma, cochlear anatomy, or other factors, predispose some individuals to showing higher EI values in apical or mid-cochlear regions.

Electrical stimulation is known to electro-chemically effect the endo-cochlear environment. When charge is delivered within safe tolerances the predominant mechanisms are ionic transfer and platinum hydrogen plating (Brummer and Turner, 1977). These processes are safe and reversible when bi-phasic charge-balanced pulses are used. It has been suggested that such charge delivery mediates the process of protein adsorption onto platinum electrodes and can affect the organization and density of the fibrotic capsule (Newbold et al., 2010). It is well documented that electrode deactivation contributes to EI increase, so ideally clinicians would access objective evidence before making electrode deactivations that make future reactivation more difficult. Neuburger et al. (2009) presents further evidence of the effect of electrical stimulation on impedance. They observed cases of increasing EI in CI users with high rates of stimulation, which necessitate short pulse-width and high current to produce the desired perceived loudness. A therapeutic intervention involving increased pulse-width along with antibiotics and steroids proved effective at significantly reducing EI. The author suggests that the original EI increase could be caused by the occurrence of out-of-compliance charge delivery leading to slight asymmetries in bi-phasic pulses. Early detection of increasing impedance could therefore be clinically important: it will inform stimulus parameter adjustments, which could lower impedance levels before they cause voltage compliance problems.

Recent work has identified improved preservation of spiral ganglion neurones after dexamethasone elution in chronically stimulated animals (Scheper et al., 2017). Another study of dexamethasone eluting CI electrodes in guinea pigs showed significant reductions in fibrotic tissue and EI compared to no-steroid controls (Wilk et al., 2016). A complementary result was shown in humans where the cochlea was perfused with the steroid triamcinolone; long-term EI levels were significantly lower in the treatment group compared to controls (De Ceulaer et al., 2003). Systemic delivery of the steroid methylprednisolone in another study did not reduce EI spikes (Choi et al., 2017), which suggests the anti-inflammatory action of steroids is most effective when topically administered. It would be interesting to study the benefit of steroid based intervention that is directed by the outlier-labeling rule used here.

Our analysis of the proportion of deactivated electrodes in children and adults was quite telling. Generally, both age groups showed a pattern of electrode deactivation primarily at basal electrodes. However, the reasons for deactivations were overwhelmingly patient feedback from adults whereas the most common reason in children was extra-cochlear position. In addition, deactivation was less common in children than in adults. One possible explanation for this was that clinical decisions about deactivations are more cautious with children, or, rather, that adult feedback to clinicians does lead to choice of deactivation of electrodes with more fibrous tissue grown/higher EI (e.g., primarily basal). This begs the question of whether choice to deactivate electrodes is optimal, and in particular whether the smaller proportion of basal electrode deactivations among children in particular is clinically appropriate or whether deactivation of basal electrodes in adults is excessive. Cross-referencing with the outlier method of EI analysis and other methods noted above could help to determine the answer to these questions. Alternatively, it may be that some differences in etiology and/or anatomy pre-dispose the child’s cochlea to be more susceptible to other types of problem (e.g., non-auditory stimulation).

## Conclusion

An important outcome of this work is the insight gained from applying a custom analysis protocol to existing clinical data. Our approach was to characterize sample-wide trends and apply an outlier detection rule that could improve our early detection of sub-optimal performance. A key benefit of using this method alongside manufacturer-specific proprietary telemetry systems is the sensitivity to changes of lower magnitude that may be associated with performance. This offers clinicians and researchers working in neuroprosthetics a method for interrogating their existing population data to identify incremental changes in device behavior, without extra financial, technical or ethical burden.

Our first question addressed the trend of impedance change over time for different electrode positions. The results showed that electrodes exhibit distinct trends of impedance evolution over 5 years. In the adult group growth in the basal electrodes contrasted with reduction for apical electrodes. The results also describe the range of the adult and pediatric dataset, which provides useful insights into individual variability. One reason for characterizing the EI trends over time was to improve interpretation of any individual deviation from the normative range. We asked how many individuals show statistically raised EI. The main analysis showed 8% of adults and 5% of children exhibited raised EI levels compared to the sample distribution. These cases were detected using a statistical outlier-labeling rule, which could be used to inform electrode deactivations with improved objectivity. Indeed, our findings show that clinical decisions to deactivate electrodes for adults were most commonly informed by patient subjective reports. The fact that adults had proportionally more electrodes deactivated than children may be caused by differences in capacity and confidence for verbal communication. The method used here to detect raised impedance in individuals of a clinical population may offer an opportunity to activate or deactivate electrodes long before the current device-specific floor or ceiling levels are reached. We determine that the information extracted from populations of users can be used alongside subjective reports to inform clinical management of individual patients. More work is needed to explore the sensitivity of this method as a biomarker of CI performance decrement.

The immediate benefit of these methods and findings is to give clinicians fresh insight into their existing data. The increasing size and accessibility of clinical datasets presents an opportunity to professionals working with neuroprosthetics. Population-wide norms can be used to better interpret measurements from individual patients. The aim is to personalize clinical management to improve the function and biocompatibility of the implant interface over a user’s lifetime.

## Author Contributions

AS conceived the research idea in conversation with TN and CV (academic supervisory team), performed the data collection, design of analysis, and literature review, and wrote the manuscript with guidance from TN and CV. ER wrote the custom MathWorks MATLAB code used for main analyses (as detailed in section “Materials and Methods”) and automated generation of Figures 2–4, 5, 7, 8 with contribution from AS. AS, ER, TN, and CV performed the final manuscript editing and proofreading process with equal contribution.

## Conflict of Interest Statement

The authors declare that the research was conducted in the absence of any commercial or financial relationships that could be construed as a potential conflict of interest.
