# Supplementary material for: Exploiting Routine Clinical Measures to Inform Strategies for Better Hearing Performance in Cochlear Implant Users
Source: Front Neurosci. 2019 Jan 15;12:1048. doi: 10.3389/fnins.2018.01048 (PMC6340939; doi:10.3389/fnins.2018.01048)
Supplement: Supplementary file 1 [file Data_Sheet_1.docx]

**Supplementary figures**


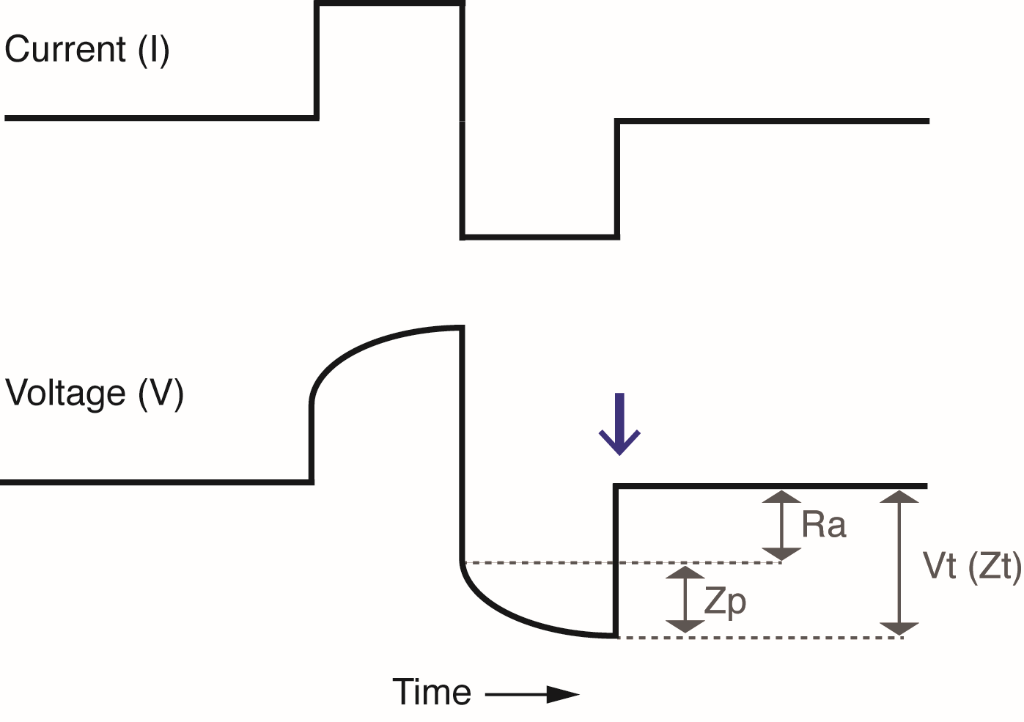


**Figure 1:** Input current and resulting voltage across active and reference electrode during monopolar impedance measurement. The blue arrow indicates the measurement time. $V_{t}$, Total voltage; $Z_{t}$, Total impedance; $Z_{p}$, Polarization impedance; $R_{a}$, Access resistance.

**
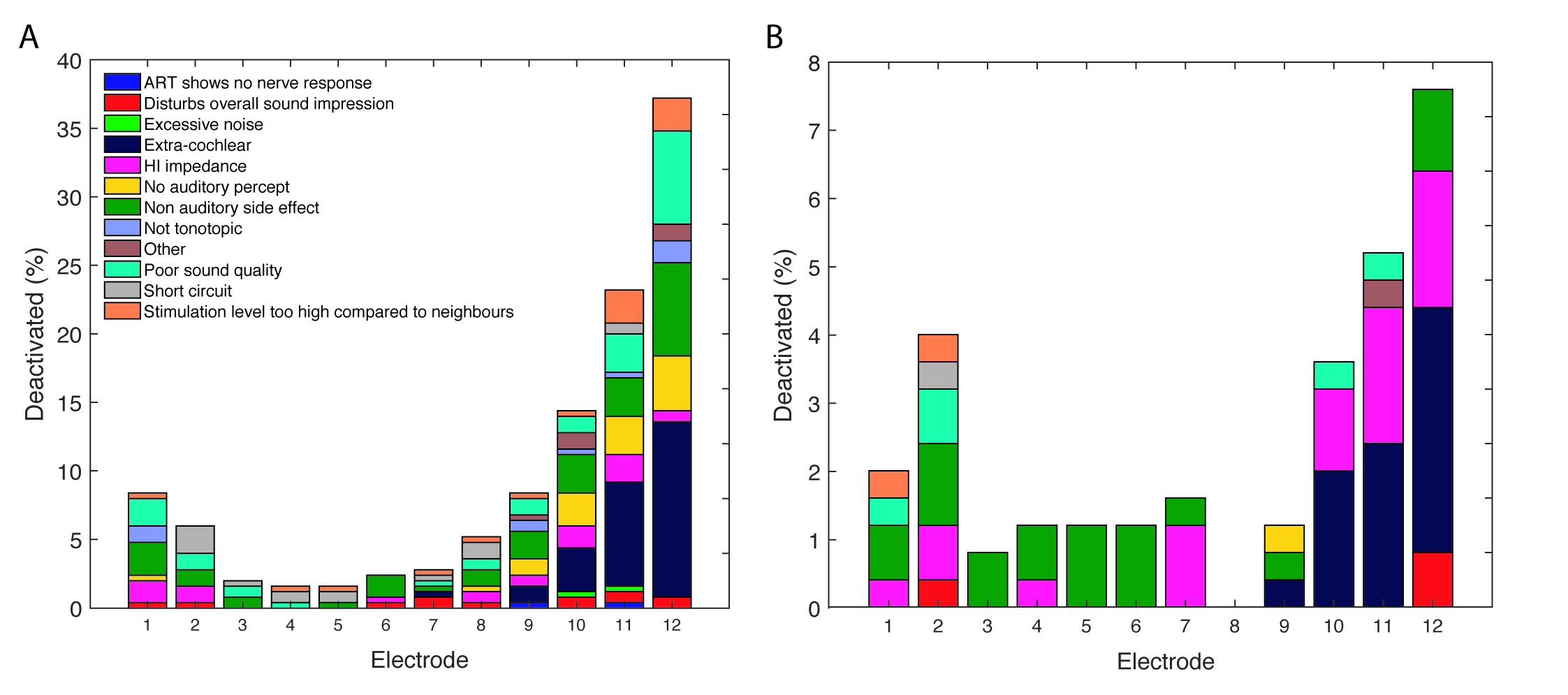
**

**Figure 2:** Count of reasons for deactivation (Other: 'to see if whistling goes', 'small dynamic range', 'poor loudness growth on ART', 'disabled', 'Disabled to increase rate', 'Disabled to increase channel separation', 'Disabled in the imported CIStudio+ Map'


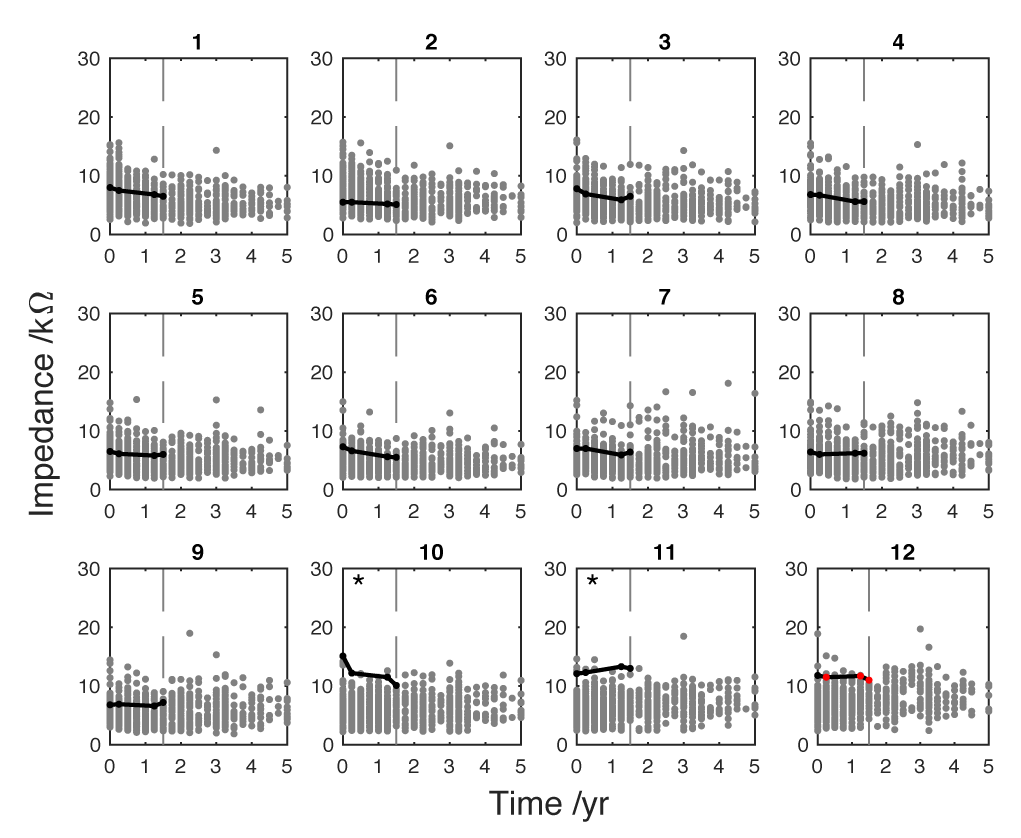


**Figure 3:** An individual adult case showing EI. Case shows 1.5 years CI use indicated by vertical dotted line. Electrode marked by * met the SEI criteria which indicates high EI compared to the sample distribution. Red dots indicate EI data points measured at deactivated electrodes (not included in SEI detection).


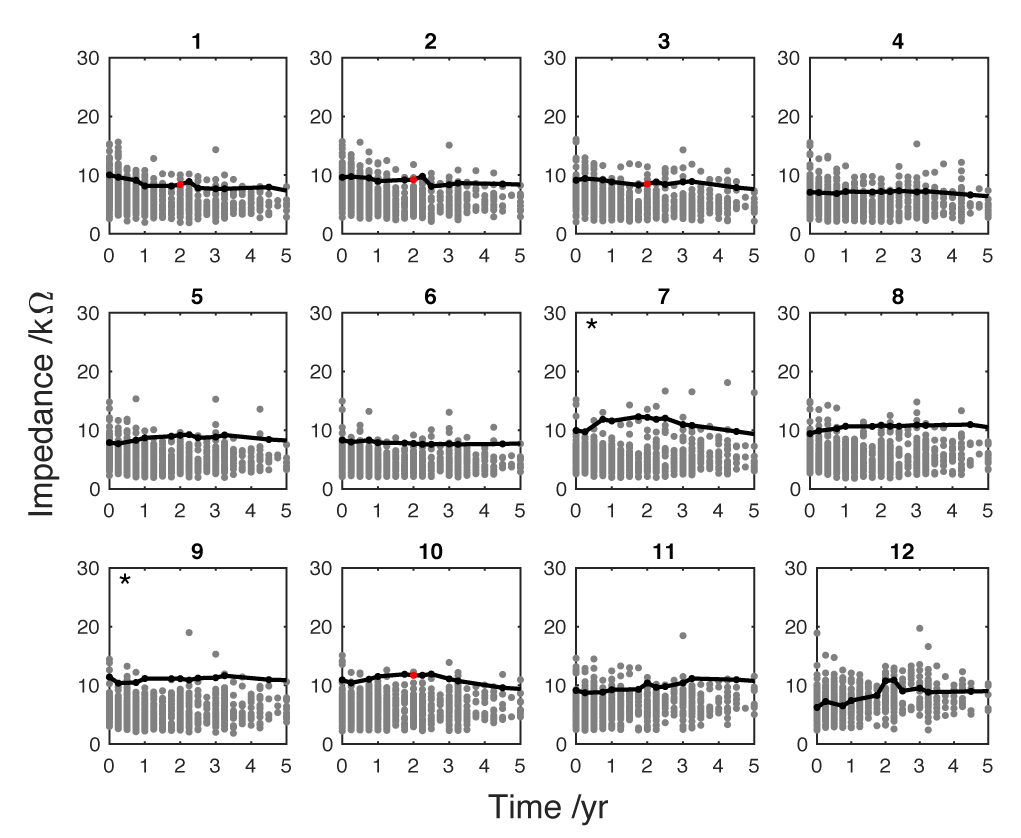


**Figure 4:** An individual adult case showing EI. Case shows 5 years CI use indicated by vertical dotted line. Electrode marked by * met the SEI criteria which indicates high EI compared to the sample distribution. Red dots indicate EI data points measured at deactivated electrodes (not included in SEI detection).


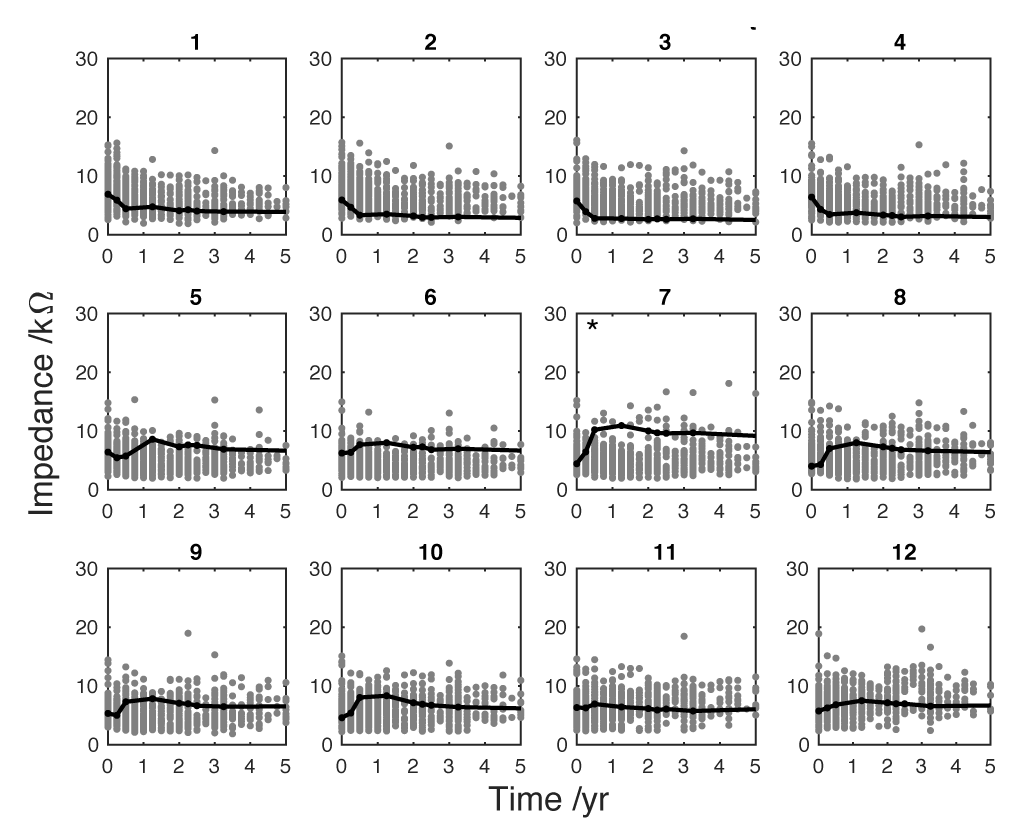


**Figure 5:** An individual adult case showing EI. Case shows 5 years CI use indicated by vertical dotted line. Electrode marked by * met the SEI criteria which indicates high EI compared to the sample distribution.


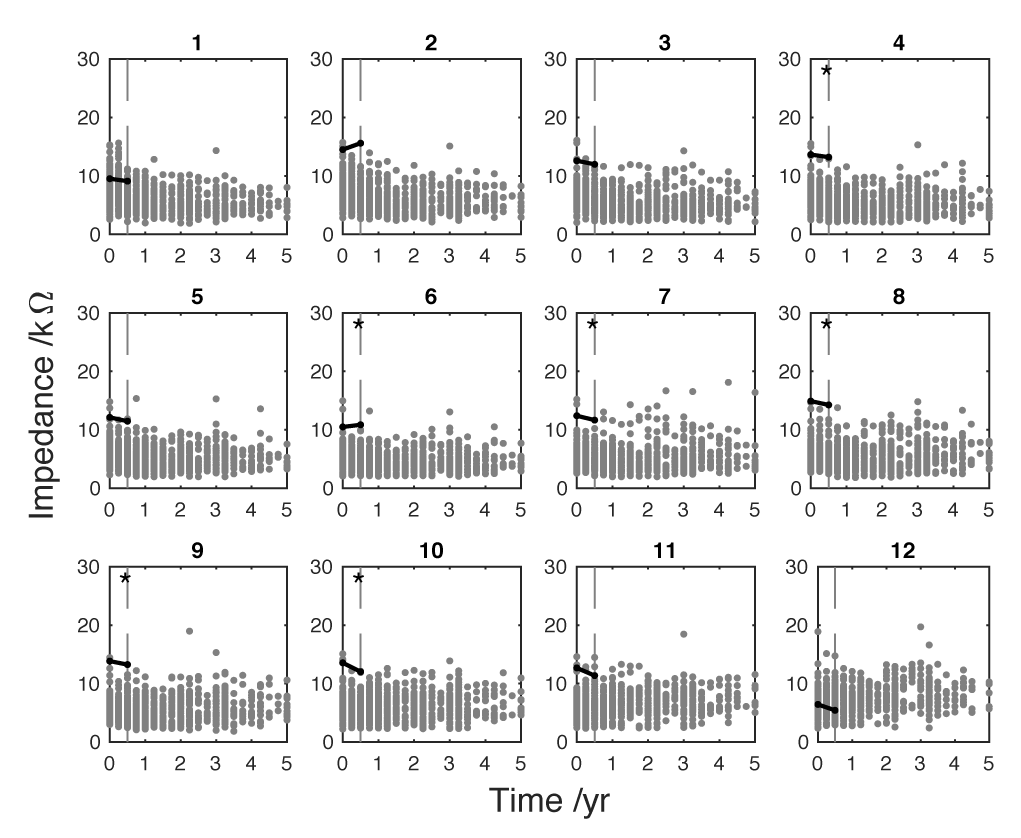


**Figure 6:** An individual adult case showing EI. Case shows 0.5 years CI use indicated by vertical dotted line. Electrode marked by * met the SEI criteria which indicates high EI compared to the sample distribution.


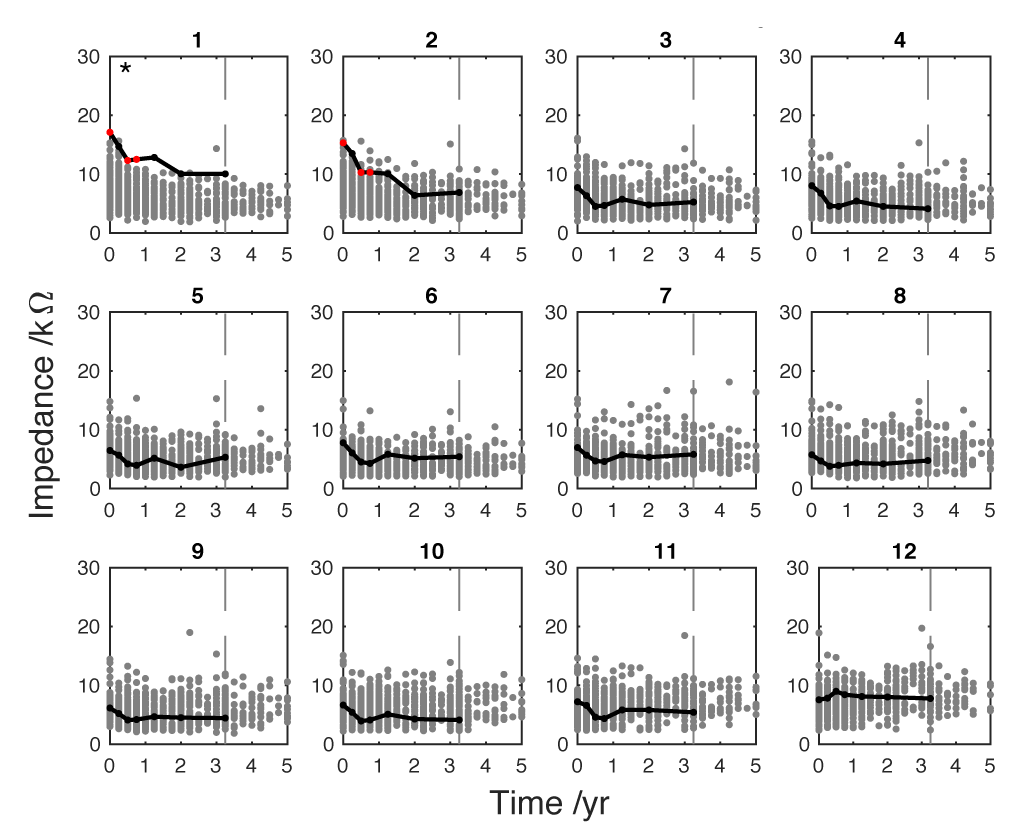


**Figure 7:** An individual adult case showing EI. Case shows 3.25 years CI use indicated by vertical dotted line. Electrode marked by * met the SEI criteria which indicates high EI compared to the sample distribution. Red dots indicate EI data points measured at deactivated electrodes (not included in SEI detection).


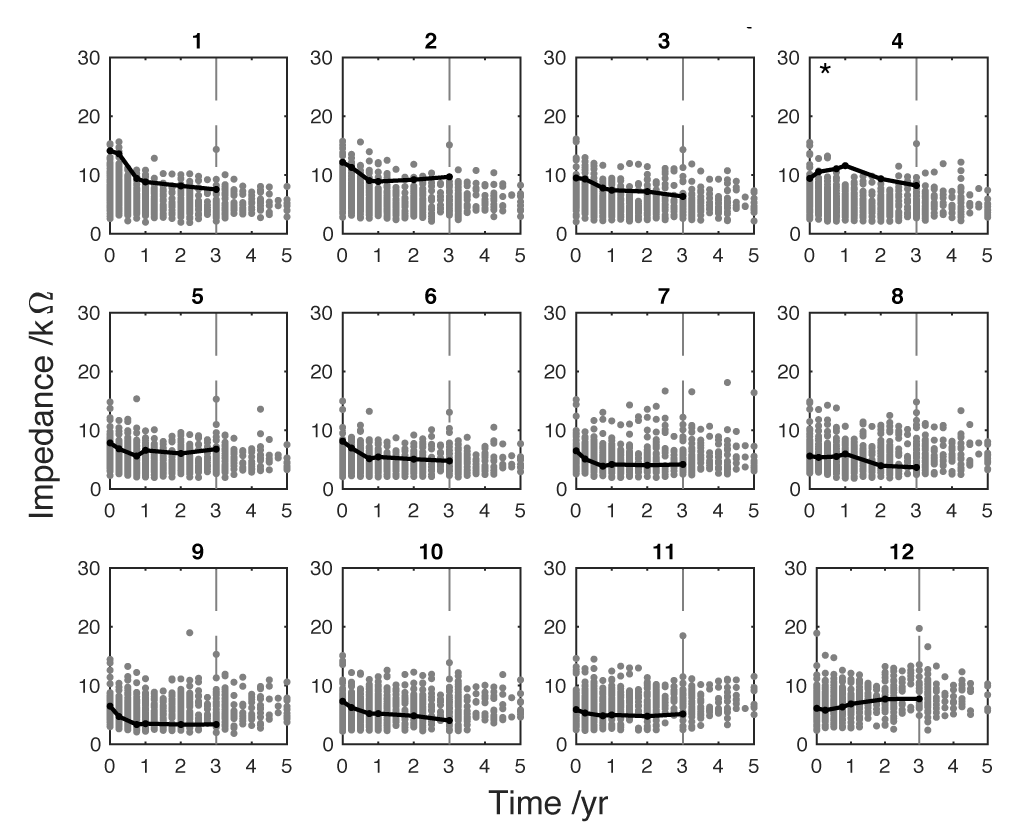


**Figure 8:** An individual adult case showing EI. Case shows 3 years CI use indicated by vertical dotted line. Electrode marked by * met the SEI criteria which indicates high EI compared to the sample distribution.


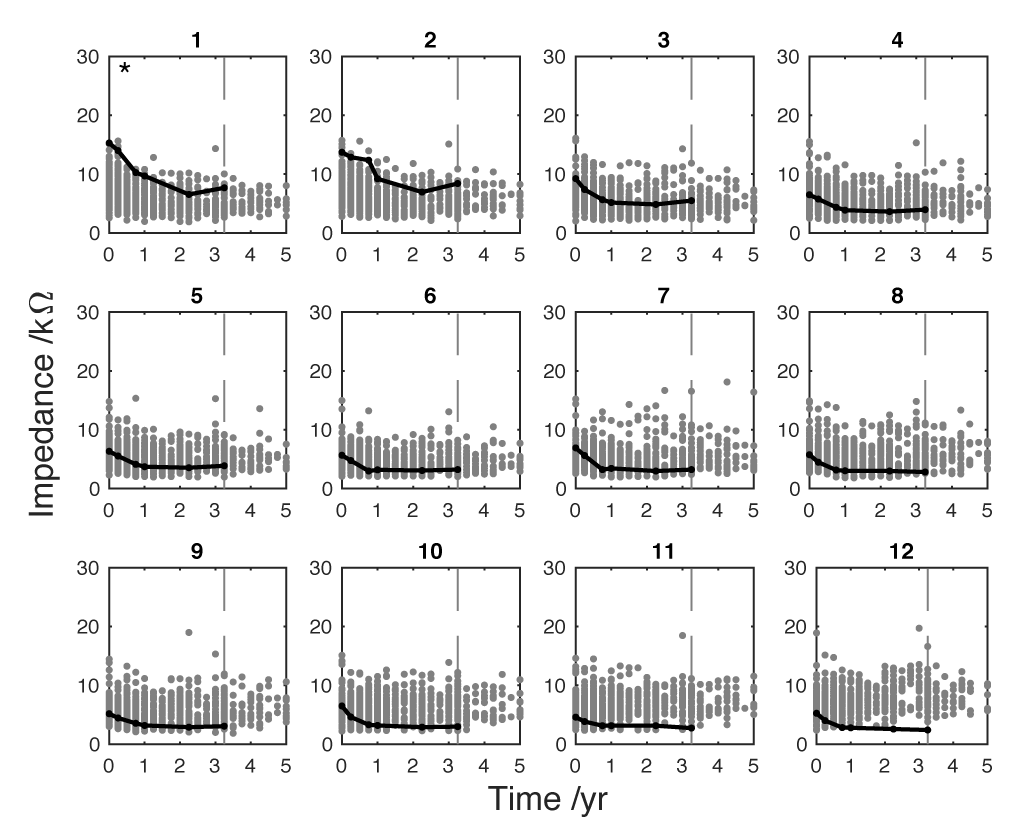


**Figure 9:** An individual adult case showing EI. Case shows 3.25 years CI use indicated by vertical dotted line. Electrode marked by * met the SEI criteria which indicates high EI compared to the sample distribution.


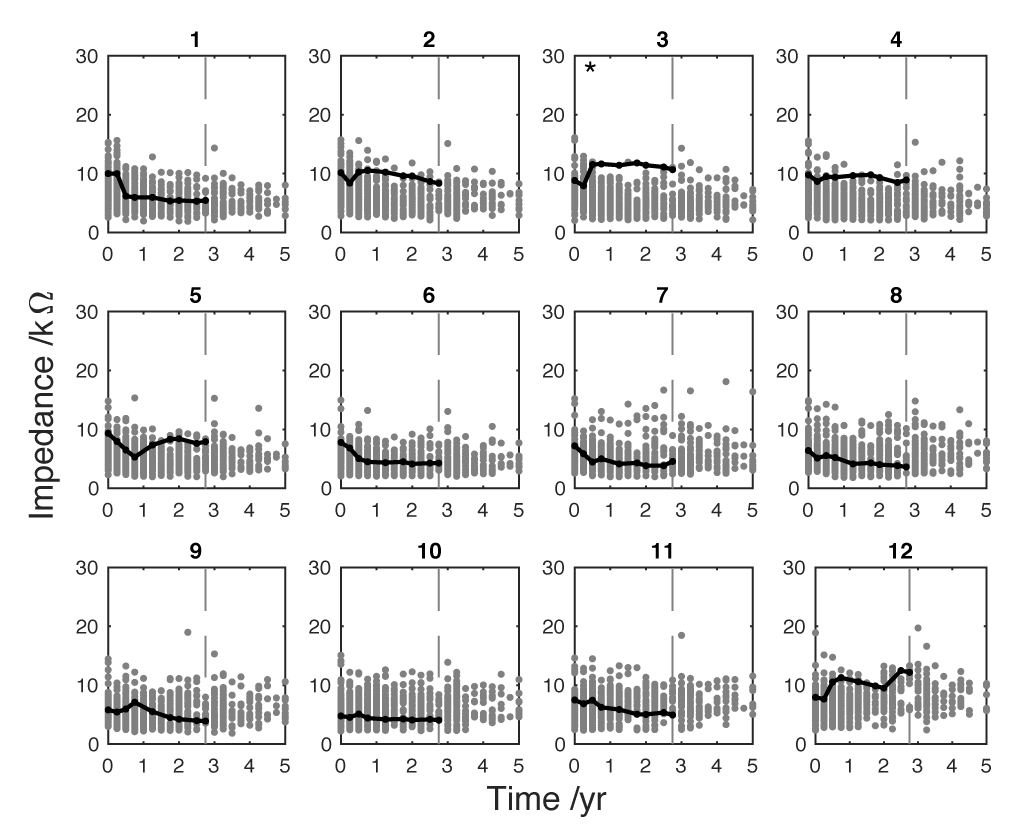


**Figure 10:** An individual adult case showing EI. Case shows 2.75 years CI use indicated by vertical dotted line. Electrode marked by * met the SEI criteria which indicates high EI compared to the sample distribution.


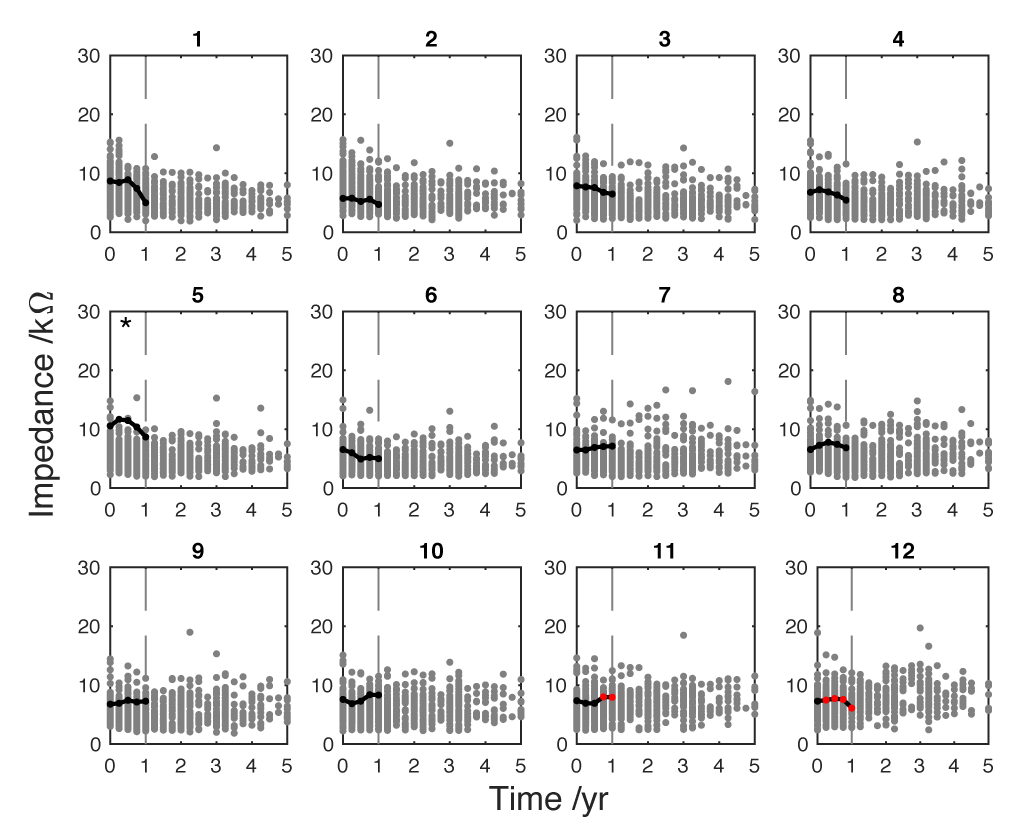


**Figure 11:** An individual adult case showing EI. Case shows 1 years CI use indicated by vertical dotted line. Electrode marked by * met the SEI criteria which indicates high EI compared to the sample distribution. Red dots indicate EI data points measured at deactivated electrodes (not included in SEI detection).


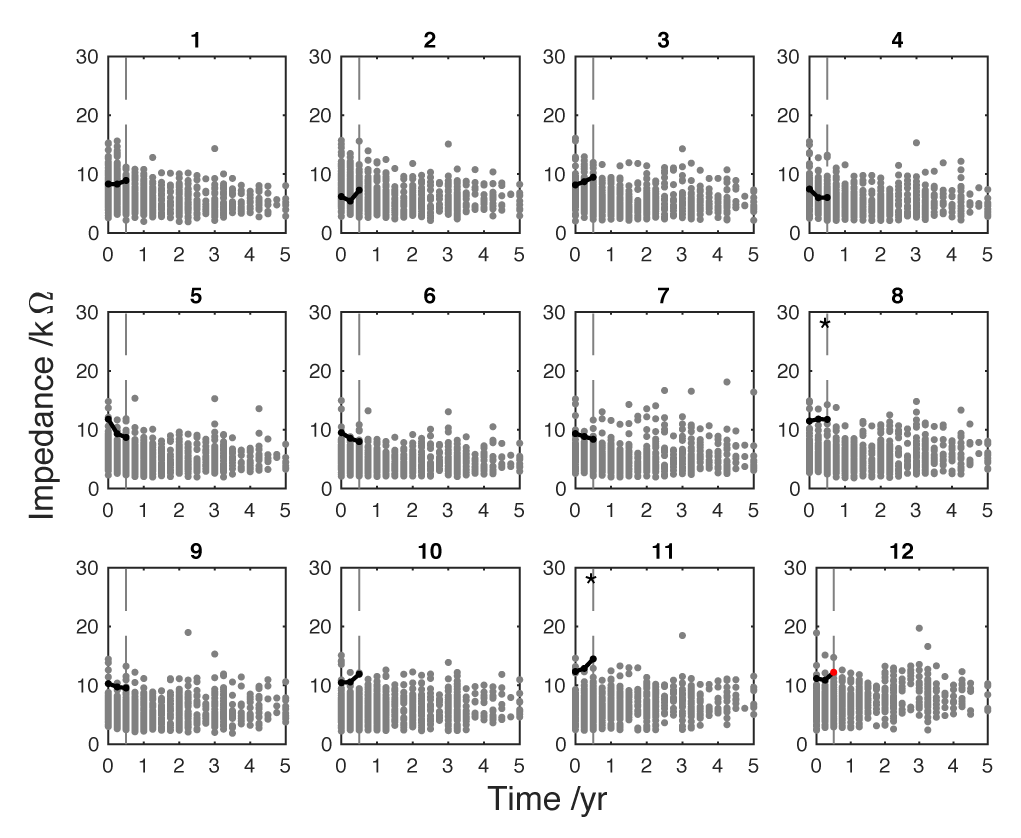


**Figure 12:** An individual adult case showing EI. Case shows 0.5 years CI use indicated by vertical dotted line. Electrode marked by * met the SEI criteria which indicates high EI compared to the sample distribution. Red dots indicate EI data points measured at deactivated electrodes (not included in SEI detection).


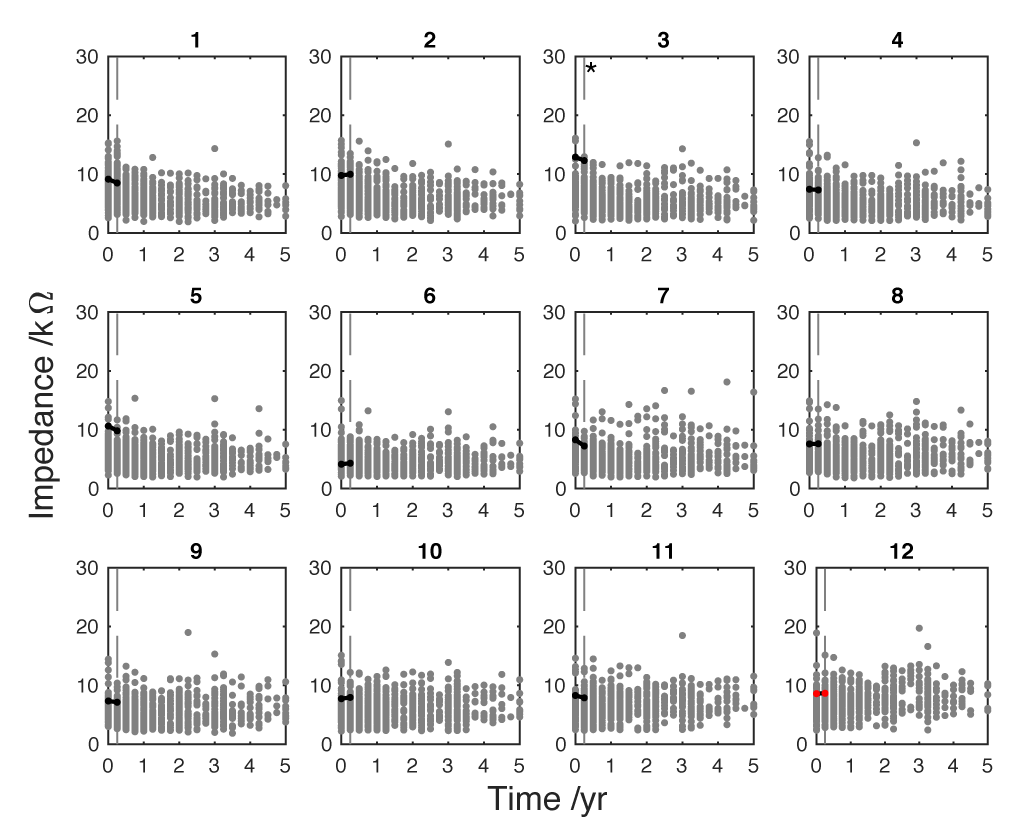


**Figure 13:** An individual adult case showing EI. Case shows 0.25 years CI use indicated by vertical dotted line. Electrode marked by * met the SEI criteria which indicates high EI compared to the sample distribution. Red dots indicate EI data points measured at deactivated electrodes (not included in SEI detection).


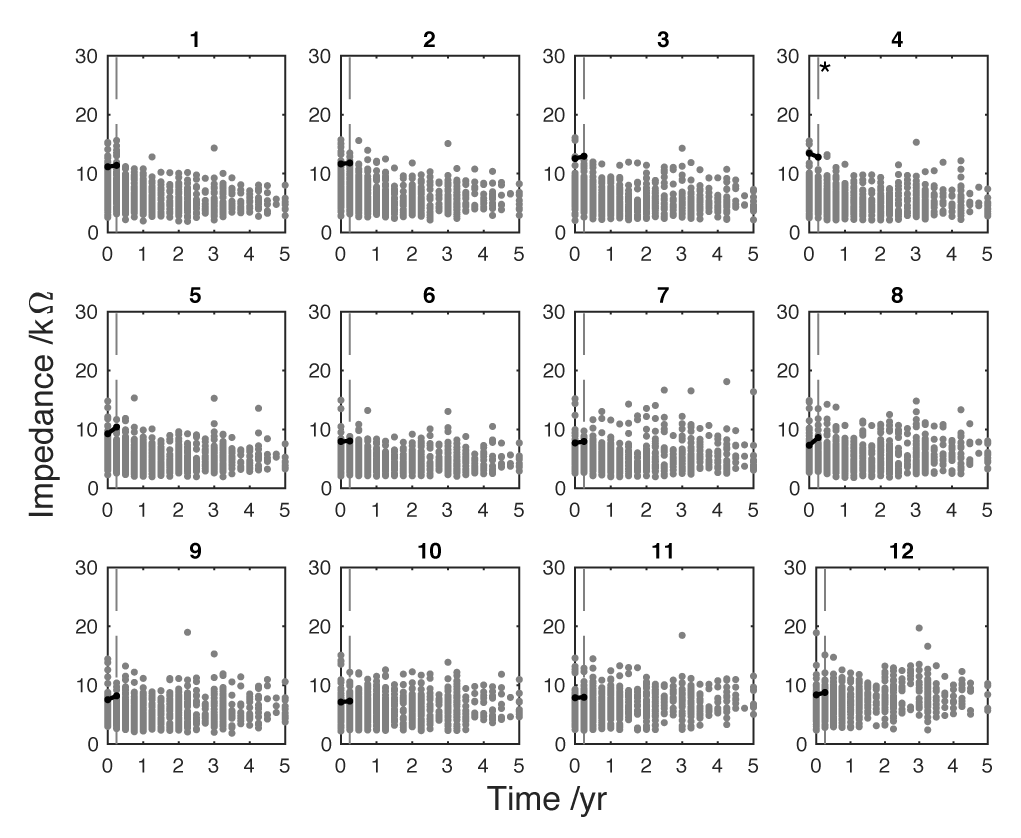


**Figure 14:** An individual adult case showing EI. Case shows 0.25 years CI use indicated by vertical dotted line. Electrode marked by * met the SEI criteria which indicates high EI compared to the sample distribution.


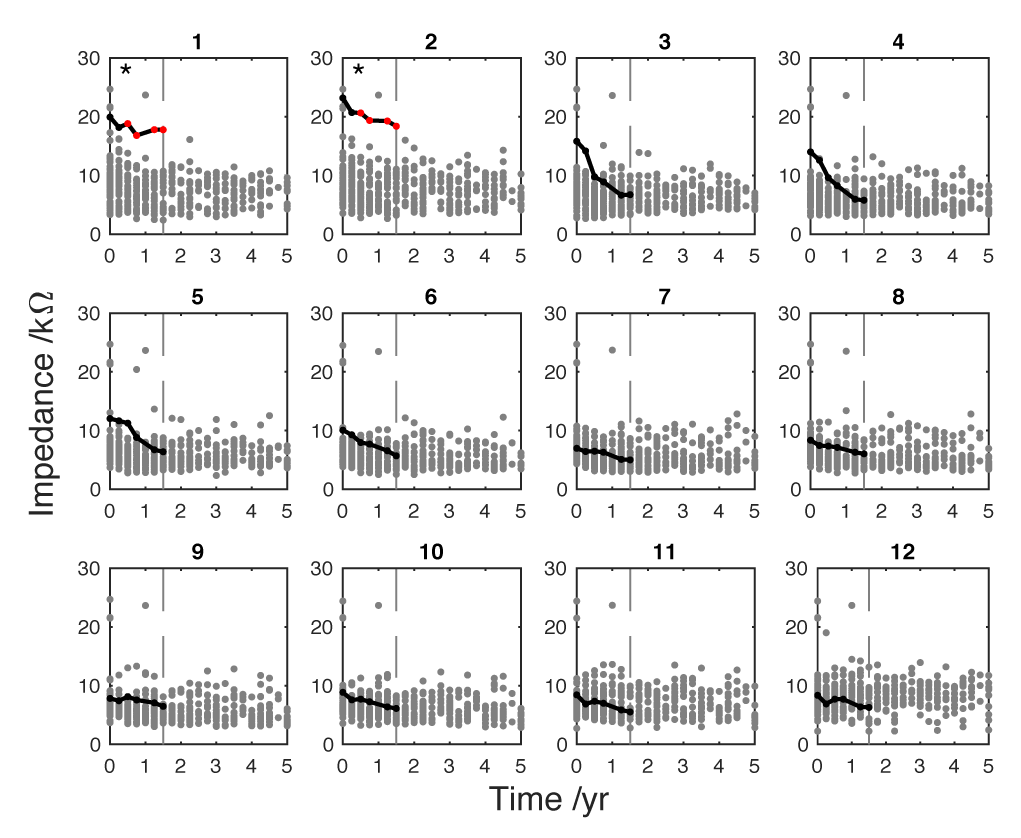


**Figure 15:** An individual paediatric case showing EI. Case shows 1.5 years CI use indicated by vertical dotted line. Electrode marked by * met the SEI criteria which indicates high EI compared to the sample distribution. Red dots indicate EI data points measured at deactivated electrodes (not included in SEI detection).
